# Is concern for gynaecological alarm symptoms associated with healthcare-seeking? A Danish population-based cross-sectional study

**DOI:** 10.1186/s12889-021-12389-x

**Published:** 2022-01-06

**Authors:** Anja Schmidt Vejlgaard, Sanne Rasmussen, Peter Fentz Haastrup, Dorte Ejg Jarbøl, Kirubakaran Balasubramaniam

**Affiliations:** Research Unit of General Practice, Department of Public Health, University of Southern Denmark, J.B. Winsløws Vej 9A, DK5000 Odense C, Denmark

**Keywords:** Primary healthcare, Gynaecology, Alarm symptoms, Women, Healthcare-seeking behaviour, Concern

## Abstract

**Background:**

Diagnosing cancer at an early stage increases survival, and for most gynaecological cancers the diagnostic pathway is initiated, when women seek medical attention with symptoms. As many factors influence healthcare-seeking, knowledge about these factors is important. Concern can act as a barrier or a trigger for women experiencing gynaecological alarm symptoms. This study aimed to examine whether concern for the symptom or the current health was associated with healthcare-seeking among women with gynaecological alarm symptoms.

**Methods:**

Some 100,000 randomly selected Danish citizens were invited to a national web-based survey. The questionnaire included items regarding symptom experiences, healthcare-seeking and concern for the experienced symptoms and current health. This study included 5019 women with self-reported gynaecological alarm symptoms (pelvic pain, pain during intercourse, bleeding during intercourse and postmenopausal bleeding). Concern was reported on a 5-point Likert scale from ‘not at all’ to ‘extremely’. Data were analysed using multivariate logistic regression models.

**Results:**

Women who were ‘extremely’ concerned about a gynaecological alarm symptom had two to six times higher odds of reporting healthcare-seeking compared to women who were ‘not at all’ concerned. Symptom concern was associated with higher odds of healthcare-seeking for all four gynaecological alarm symptoms and the odds increased with increasing levels of concern. Additionally, concern for current health was associated with higher odds of healthcare-seeking. Concern for current health as expressed by others was positively associated with healthcare-seeking but had only minor influence on the association between concern for current health and healthcare-seeking.

**Conclusions:**

Concern for a gynaecological alarm symptom and for current health was positively associated with healthcare-seeking. The results can be used for future informational health campaigns targeting individuals at risk of postponing warranted healthcare-seeking.

## Background

Cancer, including gynaecological cancers, is a major cause of mortality worldwide [1]. Diagnosing cancer at an early stage increases chances of curative treatment and reduces morbidity and mortality [2, 3]. Screening for cervical cancer has been introduced to promote earlier diagnosis [4], but the vast majority of gynaecological cancer cases are diagnosed based on symptoms, and in Denmark no screening programs exist for ovarian and endometrial cancer. The symptoms of gynaecological cancer, reported as alarm symptoms, are often non-specific and frequently experienced among women in all age groups, but in particular among younger women [5, 6], but far from every symptom experience leads to healthcare-seeking [5–7]. Knowledge about barriers and triggers for healthcare-seeking is important to understand the healthcare-seeking behaviour and improve early diagnosis. Previous studies have identified barriers for healthcare-seeking with gynaecological alarm symptoms such as perceiving the symptom as benign or absence of discomfort [8]. Awareness that the symptom can be indicative of a serious underlying disease as well as symptom severity have been identified as triggers for healthcare-seeking [8, 9]. Moreover, being older than 75 years and housebound are positively associated with seeking healthcare when experiencing gynaecological alarm symptoms [10].

How concern may be associated with healthcare-seeking with gynaecological alarm symptoms has not been fully studied. One hypothetical study found that concern of a serious underlying disease would prompt timely healthcare-seeking [8] and women who were concerned about developing gynaecological cancer were more likely to seek healthcare compared to less concerned women [10]. When differentiating concern according to intensity, worry can act as a trigger and fear as a barrier for healthcare-seeking [11]. Additionally, both embarrassment and fear of cancer can be barriers for healthcare-seeking [12].

To our knowledge no previous studies have examined the association between concern for current health and healthcare-seeking in women experiencing gynaecological alarm symptoms. Further, no previous studies have examined the concern regarding the woman’s current health as expressed by the family, the social network or a medical doctor and the association with healthcare-seeking.

This study aims to examine whether concern for the symptom and for current health is associated with healthcare-seeking among women with gynaecological alarm symptoms. Secondly, it examines whether concern regarding the woman’s current health, as expressed by the family, the social network, or a medical doctor, is associated with healthcare-seeking among women experiencing gynaecological alarm symptoms.

## Method

### Study design and population

The Danish healthcare system is tax-funded and provides equal access to universal healthcare services. The general practitioner (GP) acts as gatekeeper and first-line provider in the sense that a referral from a GP is required for most office-based specialists including gynaecologists, and always for in- and outpatient hospital treatment [13]. The National Board of Health in Denmark has decided that all women 23-59 years of age should be offered a cytological screening for cervical cancer every three – five year. The women are invited to this screening by the National Board of Health and the screening procedure of pap smear itself is in the vast majority of cases performed in general practice [4].

This cross-sectional population-based study is based on data from a Danish nationwide web-based survey, the Danish Symptom Cohort (DaSC). DaSC examined symptom experiences in the Danish population, and data collection took place between June and December 2012.

To minimize the risk of misinterpretation of the questions the questionnaire was pilot- and field-tested before distribution and adjusted hereafter. A total of 100,000 adults, of whom 51,090 were women aged 20 or above, were randomly selected from the Danish Civil Registration System (CRS), comprising the entire population, and invited to participate in the survey. Invitees received a letter explaining the study and a login to a secure webpage. Non-respondents were contacted additionally two times by reminder letter and by telephone. People without internet access were invited to complete the questionnaire as a telephone interview.

Several papers regarding symptoms and healthcare-seeking in the general population have been published based on data from DaSC [14–16]. In this particular study only women reporting gynaecological alarm symptoms were included. More information about the method can be found in Rasmussen et al. [17].

### The questionnaire

The DaSC survey comprised questions about 44 predefined symptoms in a non-cancer context. Four gynaecological alarm symptoms were included in the present study: Pelvic pain, pain during intercourse, bleeding during intercourse and postmenopausal bleeding. Although not specified in the questionnaire, these symptoms can be indicative of gynaecological cancer [18]. When the term ‘the symptom’ is used in the current paper, it refers to any of these gynaecological alarm symptoms. For each of the four symptoms the following question was phrased: “*Have you experienced any of the following bodily sensations, symptoms or discomfort within the past four weeks?”* The reply options were ‘*Yes*’, ‘*No*’ or *‘Do not wish to answer’.*

To examine if the experience of a gynaecological alarm symptom led to contact with the healthcare system the question was phrased: “*Have you been in contact with your general practitioner regarding the below mentioned symptom or discomfort?”* (Yes/ no).

Concern for the experience of each of the four symptoms was examined by the following question: “*To what extent are you concerned about the following symptoms or discomfort?”* (Not at all/ slightly/ moderately/ quite a bit/ extremely).

In a separate question the respondents were asked if they were concerned for their current health in general, i.e., not related to any specific symptom experience. The following questions were used: *“To what extent are you concerned about your current health?”* (Not at all/ slightly/ moderately/ quite a bit/ extremely), *“Has a doctor expressed concern about your current health?”* (Yes/ no/ I don’t know) and *“Have people in your family or social network expressed concern about your current health?”* (Yes/ no/ I don’t know).

### Data analysis

We included women answering all the relevant questions and reporting at least one of the four gynaecological alarm symptoms. Women who were pregnant within the preceding six months were excluded from the analyses because the experience and interpretation of gynaecological symptoms might differ from non-pregnant women. Respondents using the response option *“Do not wish to answer”* to the questions regarding experience of each of the four symptoms were included in the analyses as not having the symptom. A total of 5019 women fulfilled all inclusion criteria and were included in this study.

Descriptive analyses provide an overview of the study base including the age distribution, concern about each of the four symptoms, concern for current health and concern for current health as expressed by the family, the social network, or a medical doctor.

Associations between concern for the symptom, own concern for current health, concern for current health as expressed by a medical doctor or the family/social network and healthcare-seeking were analysed using uni- and multivariate logistic regression models. For each of the four symptoms it was analysed whether age and concern for the symptom were associated with healthcare-seeking. Crude and age-adjusted odds ratios (ORs) were calculated. For women who reported at least one gynaecological alarm symptom it was analysed whether age (<40 years, 40-59 years, and 60+ years), own concern for current health and concern for current health as expressed by a medical doctor or the family/the social network were associated with healthcare-seeking. The age categories roughly reflect the menopausal transition (pre-, peri- and postmenopausal). The association between own concern for current health and healthcare-seeking were adjusted for age and concern for current health as expressed by both a medical doctor and the family/the social network.

## Results

**Fig. 1:**
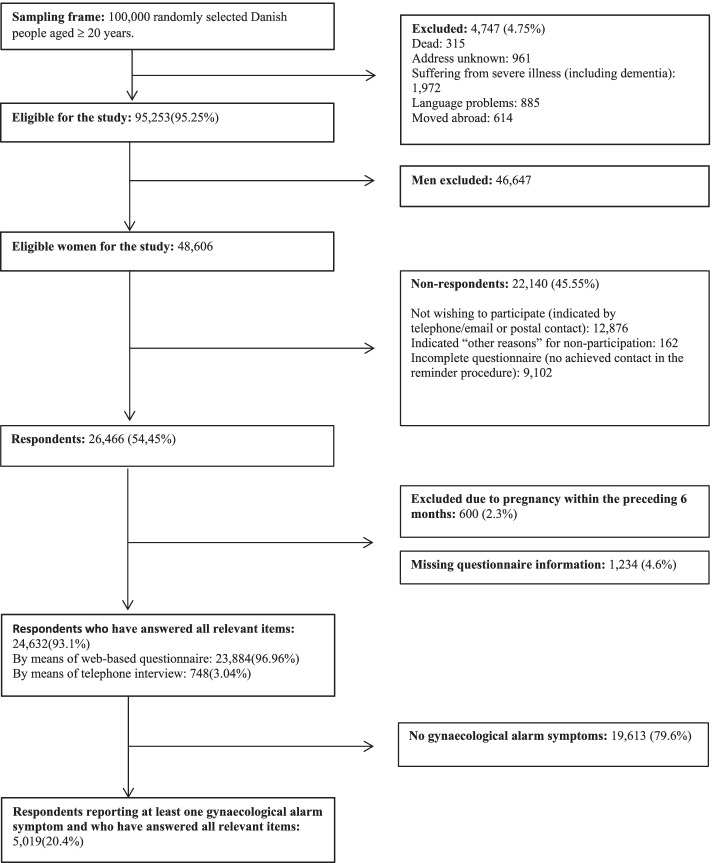
Flow of participants

Some 26,466 women answered the questionnaire, yielding a response rate of 54.5%. A total of 5,019 non-pregnant women fulfilled all inclusion criteria and were thus included in this study (Fig. 1).

Table 1 shows the descriptive data on the study population. The proportion of women who reported being ‘not at all’ concerned regarding a symptom experience ranged from 24.9% to 55.4% for pain during intercourse and postmenopausal bleeding, respectively. The proportion of women who were ‘extremely’ concerned about their current health ranged from 4.5% to 7.7% for pelvic pain and bleeding during intercourse, respectively.

**Table 1:** Descriptive data on the study population

	All women	Minimum one gynaecological alarm symptom	Pelvic pain	Pain during intercourse	Bleeding during intercourse	Postmenopausal bleeding
	n (%)	n (%)	n (%)	n (%)	n (%)	n (%)
Total:	24,632 (100.0)	5019 (100.0)	3564 (100.0)	1909 (100.0)	569 (100%)	323 (100%)
Age:						
***<****40*	6292 (25.5)	2263 (45.1)	1840 (51.6)	875 (45.8)	308 (54.1)	
*40-59*	10,283 (41.7)	2113 (42.1)	1396 (39.2)	754 (39.5)	206 (36.2)	239 (74.0)
***≥****60*	8057 (32.7)	643 (12.8)	328 (9.2)	280 (14.7)	55 (9.7)	84 (26.0)
Concern for the symptom:						
*Not at all*			1254 (35.2)	475 (24.9)	156 (27.4)	179 (55.4)
*Slightly*			1185 (33.2)	678 (35.5)	206 (36.2)	73 (22.6)
*Moderate*			624 (17.5)	356 (18.6)	94 (16.5)	33 (10.2)
*Quite a bit*			341 (9.6)	254 (13.3)	69 (12.1)	21 (6.5)
*Extremely*			160 (4.5)	146 (7.6)	44 (7.7)	17 (5.3)
Concern about current health:						
*Not at all*	9190 (37.3)	1264 (25.2)				
*Slightly*	10,309 (41.9)	2307 (46.0)				
*Moderate*	3245 (13.2)	829 (16.5)				
*Quite a bit*	1496 (6.1)	483 (9.6)				
*Extremely*	392 (1.6)	136 (2.7)				
Doctor concern about current health:						
*Yes*	2111 (8.6)	538 (10.7)				
*No*	2017 (81.9)	3904 (77.8)				
*I don’t know*	2350 (9.5)	577 (11.5)				
Family or social network concern about current health:						
*Yes*	4903 (19.9)	1417 (28.2)				
*No*	18,158 (73.7)	3253 (64.8)				
*I don’t know*	1571 (6.4)	349 (7.0)				

Among women reporting at least one gynaecological alarm symptom 9.6% and 2.7% of the women were ‘quite a bit’ and ‘extremely’ concerned about their current health, respectively, and 10.7% and 28.2%, reported that a medical doctor or the family/the social network had expressed concern for their current health, respectively. For all female respondents (*n* = 24,632), irrespective of symptom experience, a total of 8.6% and 19.9% reported that a medical doctor or the family/the social network had expressed concern about their current health, respectively (Table 1).

### Concern for the experience of gynaecological alarm symptoms and healthcare-seeking (Tables 2 and 3)

Table 2 shows proportion of women who contacted the GP in the different age categories and concern for the symptom, stratified by the four gynaecological alarm symptoms.

**Table 2:** The proportion of women who seek healthcare according to age- and concern group

	Healthcare-seeking for pelvic pain	Healthcare-seeking for pain during intercourse	Healthcare-seeking for bleeding during intercourse	Healthcare-seeking for postmenopausal bleeding
	n (%)	n (%)	n (%)	n (%)
Total:	907 (25.4%)	511 (26.8%)	177 (31.1%)	107 (33.1%)
Age:				
*<40*	442 (24.0)	214 (24.5)	85 (27.6)	
*40-59*	350 (25.1)	216 (28.6)	67 (32.5)	72 (30.1)
***≥****60*	115 (35.1)	81 (28.9)	25 (45.5)	35 (41.7)
Concern for the symptom:				
*Not at all*	133 (10.6)	92 (19.4)	28 (17.9)	37 (20.7)
*Slightly*	254 (21.4)	118 (17.4)	55 (26.7)	24 (32.9)
*Moderate*	251 (40.2)	116 (32.6)	36 (38.3)	15 (45.5)
*Quite a bit*	160 (46.9)	101 (39.8)	35 (50.7)	17 (81.0)
*Extremely*	109 (68.1)	84 (57.5)	23 (52.3)	14 (82.4)

**Table 3:** The association between concern for the symptom and healthcare-seeking, stratified by gynaecological alarm symptoms

	Healthcare-seeking for pelvic pain		Healthcare-seeking for pain during intercourse		Healthcare-seeking for bleeding during intercourse		Healthcare-seeking for postmenopausal bleeding	
	Crude OR (95%-CI)	Adjusted OR ^a^ (95%-CI)	Crude OR (95%-CI)	Adjusted OR ^a^ (95%-CI)	Crude OR (95%-CI)	Adjusted OR ^a^ (95%-CI)	Crude OR (95%-CI)	Adjusted OR ^a^ (95%-CI)
Age:								
*<40*	1		1		1			
*40-59*	1.06 (0.90;1.24)		1.24 (0.99;1.55)		1.26 (0.86;1.86)		1	
***≥****60*	1.71 (1.33;2.19)		1.26 (0.93;1.70)		2.19 (1.22;3.93)		1.66 (0.99;2.77)	
Concern for the symptom:								
*Not at all*	1	1	1	1	1	1	1	1
*Slightly*	2.30 (1.83;2.89)	2.24 (1.78;2.82)	0.88 (0.65;1.19)	0,87 (0.64;1.17)	1.67 (1.00;2.78)	1.63 (0.97;2.73)	1.88 (1.02;3.45)	2.08 (1.12;3.88)
*Moderate*	5.67 (4.46;7.21)	5.51 (4.32;7.02)	2.01 (1.46;2.76)	2.00 (1.45;2.75)	2.84 (1.58;5.08)	2.93 (1.63;5.30)	3.20 (1.47;6.94)	3.24 (1.48;7.09)
*Quite a bit*	7.45 (5.64;9.84)	7.28 (5.50;9.63)	2.75 (1.96;3.86)	2.78 (1.98;3.90)	4.71 (2.52;8.79)	5.17 (2.74;9.76)	16.31 (5.18;51.39)	17.63 (5.53;56.14)
*Extremely*	18.01 (12.34;26.29)	17.78 (12.18;25.97)	5.64 (3.78;8.41)	5.88 (3.94;8.79)	5.01 (2.44;10.28)	5.73 (2.75;11.94)	17.91 (4.89;65.61)	18.14 (4.91;67.04)

Bold denotes significant results

*OR* Odds Ratio, *CI* Confidence Interval

^a^ Adjusted for age

Table 3 shows the crude and adjusted odds ratios for the association between concern for each of the four gynaecological alarm symptoms and GP contacts.

Among women who reported to be ‘extremely’ concerned about the symptom 52.3% and 82.4% reported that they had contacted the GP with bleeding during intercourse and postmenopausal bleeding, respectively.

Women reporting postmenopausal bleeding and being ‘extremely’ concerned about the symptom had higher odds (OR 18.14, 95%-confidence interval (CI): 4.91;67.04) of involving the GP compared to women who were ‘not at all’ concerned about the symptom. Similarly, for all four gynaecological alarm symptoms, being ‘moderate’, ‘quite a bit’ or ‘extremely’ concerned about the symptom, was associated with higher odds of GP contact compared to being ‘not at all’ concerned.

For both pelvic pain (OR 1.71, 95%-CI: 1.33;2.19) and bleeding during intercourse (OR 2.19, 95%-CI: 1.22;3.93) higher odds of involving the GP were found for women older than 60 years of age compared to women younger than 40 years.

### Concern for current health when experiencing at least one gynaecological alarm symptom and healthcare-seeking (Table 4)

Table 4 shows odds ratios for the association between age, concern for current health, concern expressed by a medical doctor or concern expressed by the family/social network and healthcare-seeking regarding at least one gynaecological alarm symptom.

**Table 4:** The association between age, concern for current health and healthcare-seeking

	Healthcare-seeking regarding minimum one gynaecological alarm symptom				
	n (%)	Crude OR (95%-CI)	Adjusted OR^a^ (95%-CI)	Adjusted OR^b^ (95%-CI)	Adjusted OR^c^ (95%-CI)
Age:					
*<40*	546 (24.1)	1			
*40-59*	577 (27.3)	1.18 (1.03;1.35)			
***≥****60*	221 (34.4)	1.65 (1.36;1.99)			
Concern about current health:					
*Not at all*	312 (24.7)	1	1	1	1
*Slightly*	599 (26.0)	1.07 (0.91;1.25)	1.07 (0.91;1.25)	1.06 (0.90;1.24)	1.05 (0.89;1.24)
*Moderate*	230 (27.7)	1.17 (0.96;1.43)	1.15 (0.95;1.41)	1.13 (0.92;1.39)	1.13 (0.91;1.40)
*Quite a bit*	156 (32.3)	1.46 (1.16;1.83)	1.43 (1.14;1.80)	1.33 (1.03;1.71)	1.38 (1.06;1.78)
*Extremely*	47 (34.6)	1.61 (1.11;2.35)	1.56 (1.07;2.28)	1.45 (0.98;2.16)	1.51 (1.01;2.25)
Doctor concern about current health:					
*Yes*	181 (33.6)	1.45 (1.20;1.75)			
*No + I don’t know*	1163 (26.0)	1			
Family or social network concern about current health:					
*Yes*	417 (29.4)	1.20 (1.05;1.38)			
*No + I don’t know*	927 (25.7)	1			

^a^ Adjusted for age

^b^ Adjusted for doctor concern about current health

^c^ Adjusted for family or social network concern about current health

The analyses showed higher odds of involving the GP when being above 60 years (OR 1.65, 95%-CI: 1.36;1.99) or 40-59 years (OR 1.18, 95%-CI: 1.03;1.35) compared to being <40 years. Additionally, higher odds of healthcare-seeking were observed when concern for the woman’s current health was expressed by the family/the social network (OR 1.20, 95%-CI: 1.05;1.38) or a medical doctor (OR 1.45, 95%-CI: 1.20;1.75).

Being ‘quite a bit’ (OR 1.46, 95%-CI: 1.16;1.83) and ‘extremely’ (OR 1.61, 95%-CI: 1.11;2.35) concerned about current health was associated with higher odds of healthcare-seeking compared to being ‘not at all’ concerned. Adjusting the analyses of the association between concern for current health and healthcare-seeking for 1) age, 2) concern expressed by a medical doctor and 3) concern expressed by the family/social network only changed the ORs marginally.

## Discussion

This study examined the association between concern and healthcare-seeking among women reporting gynaecological alarm symptoms. For all four gynaecological alarm symptoms women who were concerned about the symptom were more likely to seek medical attention compared to women who were not concerned. The likelihood for healthcare-seeking was also higher for women reporting concern about their current health, although this finding was less pronounced. Concern as expressed by the family/the social network or a medical doctor had only minor influence on the association between concern for current health and healthcare-seeking.

The findings of a positive association between being concerned about a gynaecological alarm symptom and healthcare-seeking is supported by other studies. The studies encompass gynaecological alarm symptoms [8] but also previous studies concerning general symptom experiences, [19, 20] as well as cancer symptoms [21] have reported an association between healthcare-seeking and being concerned about the symptom. Some studies have, however, reported that fear of cancer or treatment may act as barriers for healthcare-seeking with gynaecological alarm symptoms [22, 23]. The conflictive findings might be because these studies are exploring the underlying cause of concern, for instance concern about cancer or death.

The results of the present study indicate that higher intensity of concern is associated with a higher OR for healthcare-seeking. Although not directly comparable, Whitaker et al. [11] found that low intensity of concern (worry) is a trigger, but higher intensity of concern (fear) is a barrier for healthcare-seeking. Their findings are based on a qualitative interview study which allows for more detailed descriptions of emotions. A systematic review found the impact of emotions on healthcare-seeking to be mixed [24]. Worrying about cancer could be a reason for contacting the doctor, having nothing to worry about was often associated with longer delay and fear among cancer patients was often associated with increased delay [24]. Another study investigating the intensity of fear found similar results with worrying having no impact on contacting the doctor, fear was a factor for longer delay and being anxious had direct impact on shortening delay [25]. In the present study women were asked about concern for the symptom, without concern being explained further. Therefore, we do not know whether feelings like worry, fear and anxiety were interpreted as concern. However, in Danish worry and concern are covered by the same word.

We found that higher age was associated with increased odds of healthcare-seeking. Previous research supports this finding. A systematic review found that women >75 years experiencing a gynaecological alarm symptom are more likely not to delay healthcare-seeking compared to women of working age [26]. When examining hypothetical healthcare-seeking both younger and premenopausal women are less likely than older and postmenopausal women to report intention to seek healthcare with gynaecological symptoms [10].

Being ‘quite a bit’ or ‘extremely’ concerned about current health was positively associated with healthcare-seeking in the present study. To our knowledge no previous studies have examined the association between concern about current health and healthcare-seeking among women with gynaecological alarm symptoms. However, other studies on gynaecological cancer found that women with concern about developing a gynaecological cancer or concern that a symptom is associated with a serious underlying disease are more likely to seek healthcare [8, 10].

Concern about current health as expressed by a medical doctor or the family/the social network was associated with healthcare-seeking in women experiencing at least one gynaecological alarm symptom. The social network being important for the healthcare-seeking behaviour is also reported in previous studies; sanctioning from the social network and encouragement from others to seek help triggered healthcare-seeking in the qualitative study by Whitaker et al. [27]. Additionally, the study by Macleod et al. comparing systematic reviews showed that women experiencing low levels of social support were less likely to seek timely healthcare with endometrial cancer [23].

The association between concern for own health and healthcare-seeking was adjusted for age and concern for current health as expressed by a medical doctor or the family/the social network. These adjustments did not change the associations remarkably, which indicates that own concern, rather than concern for current health as expressed by others, drives healthcare-seeking when experiencing at least one gynaecological alarm symptom.

A strength of the DaSC study is the high response rate of 54.5% which is similar to or higher than other surveys [5, 21]. A responder analysis showed that among respondents more were women, married or living together, were more often working, and had higher educational level and higher income, compared to non-respondents [28].

The large population-based study design enables investigation of symptom experiences in a non-cancer context. This is favourable when studying self-reported symptom experiences and healthcare-seeking behaviour retrospectively, as it reduces the risk of recall-bias. To our knowledge, this is the first large population-based study to examine the association between concern for gynaecological alarm symptoms and healthcare-seeking.

Respondents had to recall symptoms within the past four weeks. This time frame was chosen to minimize recall bias while still being a sufficient period to ensure that several symptom experiences could have occurred.

A limitation is that social desirability bias could have occurred. If women felt that being concerned and thus seeking healthcare was the appropriate reaction when experiencing one of the gynaecological alarm symptoms, they might wrongfully have reported both concern and contact to the GP. This would result in an overestimation of the association between concern and healthcare-seeking. Cancer was not mentioned in the questionnaire and respondents were anonymous which could minimize the impact of social desirability bias. The anonymity also increases the possibility that invitees with self-defined private or embarrassing symptoms completed the questionnaire which minimizes reporting bias.

The web-based questionnaire form excludes invitees without internet access, for instance elderly, resulting in a possible selection bias. This was minimized by allowing completion of the questionnaire as a telephone interview. The random selection of invitees through CRS also minimized selection bias and made the study sample representative of the Danish population.

This study showed that being concerned for a symptom increases the likelihood for healthcare-seeking. Due to the cross-sectional design of the study, we cannot demonstrate any causal relationship between being concerned for a symptom and healthcare-seeking. Further, we do not know what causes concern for the given symptom, e.g. concern for serious disease or death.

Finally, we do not know whether the level of concern was associated with GP contact. It is possible that some women had been reassured by contacting the GP and therefore were little concerned and on the other hand that some women were more concerned after GP contact because of referral for further investigation. This could be a topic of focus for future research.

This study examined concern about the symptom and concern for current health as two separate variables although they might not be independent. For instance, concern for an experienced symptom can lead to concern for the current overall health or vice versa. Moreover, we cannot conclude that concern for current health was due to the experience of gynaecological alarm symptoms. Concern for current health could occur because of other factors such as comorbidity or general health concerns.

The results from this study could indicate that some women experiencing gynecological alarm symptoms probably do not know that these symptoms can be a sign of serious underlying disease and thereby it could be relevant to seek medical care. However, we cannot gain any knowledge on the true reasons behind their healthcare-seeking behavior from this study.

Health information campaigns can be effective and provide women with the necessary knowledge that for instance postmenopausal bleeding warrants investigation. The purpose of such health information campaigns should be to inform the women to be aware of gynaecological alarm symptoms so they can seek help and still reassure them that the risk of serious disease is very low [29, 30].

Future research could focus on exploring whether concern itself is associated to diagnosis of cancer. Maybe women can distinguish serious alarm symptoms from not serious which may explain why some women seek healthcare when others do not.

## Conclusion

Being concerned about experiencing a gynaecological alarm symptom was associated with higher odds of healthcare-seeking. Likewise, being concerned for the overall health was positively associated with healthcare-seeking. The association was still statistically significant when adjusted for age, a medical doctor or family/social network expressing concern for the women’s current health, implicating that the women’s own concern for their health was the main cause for healthcare-seeking.

## Data Availability

Availability of data and materials: The datasets generated and analysed during the current study are not publicly available due to the data protection regulations of the Danish Data Protection (https://www.datatilsynet.dk/english/contact-us/), Statistics Denmark (https://www.dst.dk/en/kontakt) and the Danish Health and Medicines Authority (https://www.sst.dk/en/English/About-us/Contact-us). Access to data is strictly limited to the researchers who have obtained permission for data processing. This permission was given to the Research Unit of General Practice, Department of Public Health, University of Southern Denmark.

## Abbreviations

DaSC: The Danish Symptom Cohort
CRS: Civil Registration System
GP: General practitioner
OR: Odds ratio
CI: Confidence interval
